# Patterns of antibiotic resistance in urinary tract infections before and during the COVID-19 pandemic in Uganda and Tanzania

**DOI:** 10.1093/jacamr/dlaf038

**Published:** 2025-03-14

**Authors:** Vitus Silago, Katherine Keenan, Martha F Mushi, Catherine Kansiime, Benon Asiimwe, Benjamin Sunday, Joel Bazira, Alison Sandeman, Wilber Sabiiti, Jeremiah Seni, Matthew T G Holden, Stephen E Mshana, the HATUA and CARE Consortia, David Aanensen, Annette Aduda, Benon Asiimwe, Alison Elliott, Kathryn J Fredricks, Stephen H Gillespie, Dominique L Green, Matthew T G Holden, Catherine Kansiime, Katherine Keenan, Mike Kesby, Gibson Kibiki, John Kiiru, Andy G Lynch, John Maina, Blandina T Mmbaga, Stephen E Mshana, Martha F Mushi, Joseph R Mwanga, Stella Neema, Wilber Sabiiti, Alison Sandeman, Derek J Sloan, V Anne Smith, John Stelling

**Affiliations:** Department of Microbiology and Immunology, Weill Bugando School of Medicine, Catholic University of Health and Allied Sciences, P. O. Box 1464, Mwanza, Tanzania; Geography & Sustainable Development, School of Medicine, University of St Andrews, St Andrews KY16 9AJ, UK; Department of Microbiology and Immunology, Weill Bugando School of Medicine, Catholic University of Health and Allied Sciences, P. O. Box 1464, Mwanza, Tanzania; Department of Medical Microbiology, Makerere University, P. O. Box 7062 University Rd, Kampala, Uganda; Department of Medical Microbiology, Makerere University, P. O. Box 7062 University Rd, Kampala, Uganda; Department of Microbiology, Mbarara University of Science and Technology, P. O. Box 1410, Mbarara, Uganda; Department of Microbiology, Mbarara University of Science and Technology, P. O. Box 1410, Mbarara, Uganda; Geography & Sustainable Development, School of Medicine, University of St Andrews, St Andrews KY16 9AJ, UK; Geography & Sustainable Development, School of Medicine, University of St Andrews, St Andrews KY16 9AJ, UK; Department of Microbiology and Immunology, Weill Bugando School of Medicine, Catholic University of Health and Allied Sciences, P. O. Box 1464, Mwanza, Tanzania; Geography & Sustainable Development, School of Medicine, University of St Andrews, St Andrews KY16 9AJ, UK; Department of Microbiology and Immunology, Weill Bugando School of Medicine, Catholic University of Health and Allied Sciences, P. O. Box 1464, Mwanza, Tanzania

## Abstract

**Background:**

Increased antimicrobial use during the COVID-19 pandemic has driven antimicrobial resistance (AMR) globally, particularly in resource-limited settings. This study assessed AMR patterns in urinary tract infections (UTIs) in Uganda and Tanzania before and during the pandemic.

**Methods:**

A cross-sectional study was conducted among UTI patients at healthcare facilities in Mbarara (Uganda) and Mwanza (Tanzania) between March 2019–September 2020 and January–December 2021. Mid-stream urine samples were collected and analysed following standard procedures. AMR patterns were compared across the two periods.

**Results:**

A total of 5563 patients were enrolled from Mwanza (55.0%, *n* = 3061) and Mbarara (45.0%, *n* = 2502). The overall prevalence of microbiologically confirmed UTIs in Mwanza was 32.5% (999/3060; 95% CI: 30.9%–34.3%), raised from 30.1% (655/2180; 95% CI: 28.1%–32.0%) before to 39.1% (344/880; 95% CI: 35.8%–42.4%) during the pandemic. Whereby, the overall prevalence of microbiologically confirmed UTIs in Mbarara was 24.8% (620/2502; 95% CI: 23.1%–26.5%), decreasing from 27.5% (502/1824; 95% CI: 25.5%–29.6%) before to 17.4% (118/678; 95% CI: 14.6%–20.5%) during the pandemic. The proportion of multidrug-resistant Gram-negative bacteria (MDR-GNB) rose significantly (74% versus 83.4%, *P* = 0.01) while MDR Gram-positive bacteria (MDR-GPB) increased slightly (55.5% versus 56.7%, *P* = 0.45) in Mwanza. Conversely, MDR-GPB increased substantially (31.4% versus 51.6%, *P* = 0.09) while MDR-GNB decreased (67.3% versus 61.9%, *P* = 0.22) in Mbarara.

**Conclusions:**

This study provides critical insights into AMR trends in UTI pathogens in Tanzania and Uganda, emphasizing the need for stringent antimicrobial stewardship, requiring ongoing surveillance and targeted interventions.

## Introduction

Antimicrobial resistance (AMR) is a global health crisis that threatens the effectiveness of antimicrobial therapy. The rise of AMR particularly multidrug-resistant (MDR) bacteria, particularly in low- and middle-income countries (LMICs), poses significant challenges for clinical management and healthcare systems.^1^ The overuse and misuse of antibiotics in human and veterinary medicine, compounded by limited antimicrobial stewardship, have been central drivers of AMR.^2,3^

The COVID-19 pandemic exacerbated concerns regarding AMR due to widespread antibiotic over-prescription and use, often as prophylaxis.^4,5^ Studies have indicated a surge in antibiotic consumption during the pandemic, with nearly 70% of COVID-19 patients receiving antibiotics.^4,5^ In LMICs, such as Uganda and Tanzania, pre-pandemic studies already documented extensive antibiotic misuse, including high rates of non-prescription dispensing, incomplete dosing, and self-medication, mostly in Tanzania than in Uganda.^6–8^ These behaviours, intensified by the pandemic, may have contributed to shifting AMR patterns in bacterial pathogens responsible for urinary tract infections (UTIs), among the most common bacterial infections affecting communities worldwide. The increased use of antibiotics as empirical treatments for suspected bacterial co-infections during COVID-19 may have further exacerbated resistance trends. However, limited data exist on how these practices specifically influenced UTI-related bacterial resistance in Uganda and Tanzania. This study aimed to investigate the changes in AMR and MDR patterns in UTI-causing bacteria before and during the pandemic in these settings, providing insights for future antimicrobial stewardship interventions.

## Materials and methods

### Study design and setting

This cross-sectional study was conducted in Mwanza, Tanzania and Mbarara, Uganda, during two distinct periods: pre-pandemic (March 2019–September 2020) under the HATUA project and intra-pandemic (January–December 2021) under the CARE project. Adult patients (≥18 years) presenting with UTI symptoms at outpatient clinics and inpatient settings were enrolled from multiple healthcare facilities in both regions.

### Sample collection and laboratory analysis

Mid-stream urine samples (5–10 mL) were collected from each participant using sterile containers. Samples were transported under refrigeration to the Microbiology Research Laboratory at the Catholic University of Health and Allied Sciences, Tanzania, and to the Microbiology laboratory at Mbarara University of Science and Technology, Uganda for culture and antibiotic susceptibility testing.^9^

Urine cultures were semi-quantitatively plated on blood agar, MacConkey agar and cysteine lactose electrolyte-deficient agar. Identification of uropathogens was performed using standard biochemical methods.^10^ Antibiotic susceptibility testing followed the Kirby–Bauer disk diffusion method^11^ per Clinical and Laboratory Standards Institute guidelines.^12^ Extended-spectrum beta-lactamase (ESBL) production was confirmed using the disk combination method.^12^ MDR was defined as resistance to at least one agent in three or more antibiotic categories.^13^

### Data analysis

Patient demographic and clinical data were collected using Epicollect5 and analysed using STATA 15.0 and WHONET 2022. Categorical data were presented as proportions, while statistical comparisons used chi-square and Fisher's exact tests, with *P* values <0.05 considered significant.

## Results

### Study population and UTI prevalence

A total of 5563 patients were enrolled: 3061 from Mwanza, Tanzania, and 2502 from Mbarara, Uganda. The median age of participants was 30 years in Tanzania and 34 years in Uganda. Women constituted the majority of participants (>72% in both settings and periods).

In Tanzania, microbiologically confirmed UTIs increased significantly from 30.1% (655/2180; 95% CI: 28.1%–32.0%) pre-pandemic to 39.1% (344/880; 95% CI: 35.9%–42.4%) during the pandemic (*P* = 0.01). Conversely, in Uganda, UTI prevalence declined from 27.5% (502/1824; 95% CI: 25.5%–29.6%) to 17.4% (118/678; 95% CI: 14.7%–20.4%), *P* = 0.01).

### Bacterial isolates causing UTI

Gram-negative bacteria (GNB) were the predominant uropathogens, with *Escherichia coli* and *Klebsiella pneumoniae* as the most frequently isolated species. In Tanzania, GNB isolation increased during the pandemic, accounting for 77.2% of isolates compared with 68.1% pre-pandemic. In Uganda, GNB remained predominant but decreased slightly (79.7% to 74.4%). Among Gram-positive bacteria (GPB), *Enterococcus spp.* isolation increased in both settings (Table 1).

**Table 1.** Proportions and distributions of bacterial pathogens causing UTI before and during the COVID-19 pandemic in Tanzania and Uganda

Groups and species of bacteria pathogens		Tanzania		Uganda	
		Before COVID-19	During COVID-19	Before COVID-19	During COVID-19
		Frequency (%)	Frequency (%)	Frequency (%)	Frequency (%)
Gram-negative rods	*Escherichia coli*	285 (43.1)	160 (41.9)	263 (52.4)	70 (57.9)
	*K. pneumoniae* complex	57 (8.6)	48 (12.6)	51 (10.2)	7 (5.8)
	Miscellaneous GNB	48 (7.3)	9 (2.4)	42 (8.4)	3 (2.5)
	*Pseudomonas aeruginosa*	23 (3.5)	19 (4.9)	1 (0.2)	2 (1.7)
	*Acinetobacter* spp.	9 (1.4)	18 (4.7)	5 (1.0)	0 (0.0)
	*Enterobacter cloacae* complex	9 (1.4)	8 (2.1)	3 (0.6)	1 (0.8)
	*Proteus* spp.	8 (1.2)	4 (1.0)	15 (3.0)	1 (0.8)
	*Morganella morganii*	6 (0.9)	1 (0.3)	1 (0.2)	0 (0.0)
	*Citrobacter* spp.	4 (0.6)	4 (1.0)	1 (0.2)	4 (3.3)
	*Enterobacter aerogenes*	0 (0.0)	13 (3.4)	3 (0.6)	0 (0.0)
	*Klebsiella oxytoca*	0 (0.0)	6 (1.6)	13 (2.6)	1 (0.8)
	*Providencia* spp.	1 (0.2)	2 (0.5)	1 (0.2)	1 (0.8)
	*Pantoea agglomerans*	0 (0.0)	2 (0.5)	0 (0.0)	0 (0.0)
	*Moraxella* spp.	1 (0.2)	0 (0.0)	0 (0.0)	0 (0.0)
	*Aeromonas hydrophilia*	0 (0.0)	1 (0.3)	0 (0.0)	0 (0.0)
	*Serratia marcescens*	0 (0.0)	0 (0.0)	1 (0.2)	0 (0.0)
	Total	451 (68.1)	295 (77.2)	400 (79.7)	90 (74.4)
Gram-positive cocci	CoNS	72 (10.9)	20 (5.2)	57 (11.4)	13 (10.8)
	*Staphylococcus aureus*	62 (9.4)	23 (6.0)	34 (6.8)	6 (5.0)
	*Enterococcus* spp.	35 (5.3)	27 (7.1)	3 (0.6)	9 (7.4)
	*Streptococcus* spp.	33 (4.9)	16 (4.2)	0 (0.0)	3 (2.5)
	Miscellaneous GPB	6 (0.9)	1 (0.3)	8 (1.6)	0 (0.0)
	*Staphylococcus saprophyticus*	3 (0.5)	0 (0.0)	0 (0.0)	0 (0.0)
	Total	211 (31.9)	87 (22.8)	102 (20.3)	31 (25.6)
Overall total		662 (100)	382 (100)	502 (100)	121 (100)

### AMR trends

In Tanzania, resistance rates increased significantly for several antibiotics, including ampicillin (83.9% to 95.6%), ciprofloxacin (56.1% to 67.1%), amoxicillin-clavulanic acid (43.3% to 65.6%), ceftriaxone (36.3% to 56.9%) and ceftazidime (31.8% to 53.5%) (all *P* < 0.05). In Uganda, resistance trends were mixed, with significant reductions observed for ceftriaxone (47.7% to 29.2%, *P* < 0.05) and nitrofurantoin (24.6% to 12.4%, *P* = 0.18), (Table 2).

**Table 2.** Comparison of percentage resistance of Gram-negative bacteria causing UTIs before and during the COVID-19 pandemic in Uganda and Tanzania

Antibiotic agents		Uganda				Tanzania			
		All (N = 381-490)	Before COVID-19 (N = 381–400)	During COVID-19 (N = 90)	*P* value	All (N = 589–739)	Before COVID-19 (N = 361–4444)	During COVID-19 (N = 228–295)	*P* value
Antibiotic	Strength (µg)	% [95% CI]	% [95% CI]	% [95% CI]		% [95% CI]	% [95% CI]	%[95%CI]	
**AMP**	30	85.1 [81.6–88.1]	84.2 [80.2–87.7]	88.8 [80.3–94.5]	0.15	88.1 [85.3–90.5]	83.9 [80.1–87.4]	95.6 [92.1–97.9]	0.01
**SXT**	1.25/23.75	72.2 [67.9–76.1]	72.3 [67.6–76.6]	71.9 [61.4–80.9]	0.53	87.2 [84.4–89.8]	86.7 [82.8–90.1]	88.0 [83.4–91.7]	0.33
**CIP**	5	46.3 [41.8–50.8]	44.7 [39.8–49.8]	53.3 [42.6–63.7]	0.14	60.5 [56.9–64.1]	56.1 [51.3–60.8]	67.1 [61.4–72.4]	0.01
**AMC**	10/30	57.2 [52.6–61.7]	58.8 53.7–63.8]	50.6 [39.8–61.3]	0.16	51.8 [47.9–55.7]	43.3 [38.5–48.3]	65.6 [59.4–71.4]	0.01
**CRO**	30	44.3 [39.9–48.9]	47.7 [42.7–52.8]	29.2 [20.0–39.8]	0.04	44.4 [40.7–48.2]	36.3 [31.7–41.1]	56.9 [50.8–62.8]	0.01
**NIT**	300	22.4 [18.7–26.3]	24.6 [20.4–29.2]	12.4 [6.3–21.0]	0.18	37.1 [33.3–41.1]	32.4 [27.6–37.5]	43.8 [37.6–50.2]	0.04
**CAZ**	30	30.7 [26.5–35.0]	32.3 [27.7–37.2]	23.6 [15.2–33.8]	0.21	40.0 [36.5–43.7]	31.8 [26.8–35.6]	53.5 [47.7–59.3]	0.01
**GEN**	10	17.6 [13.8–21.9]	18.0 [13.6–23.1]	16.3 [9.4–25.5]	0.44	35.3 [31.8–38.9]	29.7 [25.5–34.2]	43.9 [38.1–49.9]	0.01
**ESBL-PE**	NA	28.6 [24.6–32.8]	28.7 [24.3–33.4]	28.1 [18.4–37.5]	0.48	32.8 [29.4–36.3]	28.6 [24.4–33.1]	39.5 [33.7–45.5]	0.04

AMP, ampicillin; SXT, trimethoprim-sulfamethoxazole; CIP, ciprofloxacin; AMC, amoxicillin-clavulanic acid; CRO, ceftriaxone; NIT, nitrofurantoin; CAZ, ceftazidime; GEN, gentamicin; ESBL-PE, extended-spectrum beta-lactamases producing Enterobacterales.

### MDR bacteria trends

In Tanzania, the prevalence of MDR-GNB increased significantly from 74.7% to 83.4% (*P* = 0.01), with common MDR patterns including penicillin, cephalosporins, quinolones and aminoglycosides. MDR-GPB prevalence rose marginally (55.5% to 56.7%). In Uganda, MDR-GNB prevalence decreased slightly (67.3% to 61.9%, *P* = 0.22), whereas MDR-GPB increased substantially (31.4% to 51.6%, *P* = 0.09), (Supplementary file, available as Supplementary data at  *JAC*  Online).

## Discussion

The findings suggest that the increased antimicrobial use during the COVID-19 pandemic^6–8^ exhibited a differential impact on AMR patterns in Tanzania and Uganda. The increased AMR and MDR rates in Tanzania are likely due to heightened antibiotic overuse, as prior studies reported widespread non-prescription antibiotic sales and incomplete antibiotic courses in this region.^7^ The increase in ESBL-producing Enterobacterales, in line with another study,^14^ further signals the growing challenge of treating UTIs in Tanzania.

In contrast, the decrease in AMR prevalence in Uganda may reflect differences in antimicrobial prescribing practices, infection control policies and population behaviours during the pandemic. The rise in MDR-GPB, particularly *Enterococcus spp.*, highlights emerging resistance concerns that require further surveillance.

The observed resistance patterns have significant implications for empirical UTI treatment in LMICs. Increased resistance to fluoroquinolones, beta-lactams and trimethoprim-sulfamethoxazole may render standard first- and second-line therapies^15^ ineffective, necessitating revised antimicrobial guidelines. These trends underscore the urgent need for increased antibiotic stewardship programmes, continuous AMR surveillance and enhanced public health interventions to mitigate the growing burden of resistant infections.

## Limitations

This study has several limitations. First, it was conducted in two urban centres, limiting generalizability to other regions. Second, the cross-sectional design precludes causal inference between pandemic-related factors and resistance trends. Third, the reliance on laboratory-confirmed UTIs excludes undiagnosed or untreated cases, potentially underestimating the true burden of AMR and MDR patterns. These factors underscore the need for further studies.

## Conclusions

This study provides critical insights into AMR trends in UTI pathogens in Tanzania and Uganda before and during the COVID-19 pandemic. However, the rise of MDR-GPB in both settings highlights an evolving resistance landscape that requires ongoing surveillance. Policymakers and healthcare providers should prioritize targeted interventions to mitigate the AMR crisis in the studied settings.

## Supplementary Material

dlaf038_Supplementary_Data

## †HATUA and CARE consortia

David Aanensen, Annette Aduda, Benon Asiimwe, Alison Elliott, Kathryn J. Fredricks, Stephen H. Gillespie, Dominique L. Green, Matthew T. G. Holden, Catherine Kansiime, Katherine Keenan, Mike Kesby, Gibson Kibiki, John Kiiru, Andy G. Lynch, John Maina, Blandina T. Mmbaga, Stephen E. Mshana, Martha F. Mushi, Joseph R. Mwanga, Stella Neema, Wilber Sabiiti, Alison Sandeman, Derek J. Sloan, V. Anne Smith and John Stelling.
